# A comprehensive, multisource database for hydrometeorological modeling of 14,425 North American watersheds

**DOI:** 10.1038/s41597-020-00583-2

**Published:** 2020-07-20

**Authors:** Richard Arsenault, François Brissette, Jean-Luc Martel, Magali Troin, Guillaume Lévesque, Jonathan Davidson-Chaput, Mariana Castañeda Gonzalez, Ali Ameli, Annie Poulin

**Affiliations:** 1 Hydrology, Climate and Climate Change Laboratory, École de technologie supérieure, 1100 Notre-Dame West st., Montreal, Quebec H3C 1K3 Canada; 2 Lasalle|NHC, 9620, Saint-Patrick st., LaSalle, Quebec H8R 1R8 Canada; 3 HydroClimat|TVT, Maison du Numérique et de l’Innovation, place Georges Pompidou, Toulon, 83 000 France; 4 Department of Earth, Ocean and Atmospheric Sciences, University of British Columbia, 2020-2207 Main Mall, Vancouver, British Columbia V6T 1Z4 Canada

**Keywords:** Hydrology, Civil engineering, Water resources

## Abstract

The Hydrometeorological Sandbox - École de technologie supérieure (HYSETS) is a rich, comprehensive and large-scale database for hydrological modelling covering 14425 watersheds in North America. The database includes data covering the period 1950–2018 depending on the type and source of data. The data include a wide array of hydrometeorological data required to perform hydrological and climate change impact studies: (1) watershed properties including boundaries, area, elevation slope, land use and other physiographic information; (2) hydrometric gauging station discharge time-series; (3) precipitation, maximum and minimum daily air temperature time-series from weather station records and from (4) the SCDNA infilled gauge meteorological dataset; (5) the NRCan and Livneh gridded interpolated products’ meteorological data; (6) ERA5 and ERA5-Land reanalysis data; and (7) the SNODAS and ERA5-Land snow water equivalent estimates. All data have been processed and averaged at the watershed scale, and provides a solid basis for hydrological modelling, climate change impact studies, model calibration assessment, regionalization method evaluation and essentially any study requiring access to large amounts of spatiotemporally varied hydrometeorological data.

## Abstract (summary)

**Table:** 

Measurement(s)	elevation • temperature • hydrological precipitation process • Fluid Discharge • climate • physiographic feature
Technology Type(s)	digital curation
Sample Characteristic - Environment	watershed
Sample Characteristic - Location	North America

Machine-accessible metadata file describing the reported data: 10.6084/m9.figshare.12600281

## Background & Summary

Adequate water resources management is an essential component of socioeconomic security and development. This is made even more critical with the increasing global population and impacts of climate change on water resources. Adequate management requires knowledge about water quantity, quality as well as spatiotemporal variability in both current and future climates^1^. The Total Quality Control saying ‘*You can’t manage what you don’t measure*’ applies perfectly to water resources management. Despite this understanding, a net decline in the number of weather and gauging stations has been observed around the world^2^. To fight this decline, and especially for gauges with long-historical records that are critical to the monitoring of a changing climate, initiatives such as crowdsourcing^3^ have been put forward. In geosciences, an answer to the problems of monitoring networks has been a steady move towards gridded datasets. Gridded datasets can provide information in regions lacking adequate coverage, but are also native to many remotely sensed, computer modeled and hybrid data sources. Gridded datasets do, however, come with potential issues. Their reliability in most cases depends on the quality of the underlying observation network. In addition, while grids are used as a convenient way to represent physical processes at the regional to global scales, environmental management is not performed at the grid scale. For water resources management, the catchment-scale is the relevant geographical entity. Therefore, relevant databases for water resources management need to provide catchment-based hydrometeorological data as well as relevant physiographic variables. Complexifying the issue, there are now several available data sources for meteorological forcing data^4,5^. Recent work^6,7^ has shown that impact model results are influenced by the choice of forcing data. Providing several relevant forcing datasets should therefore be considered important to better understand how uncertainties propagate in the water resources management chain. Finally, watershed databases should make all efforts to include as many catchments as possible. A first reason is that it’s been shown that local studies are difficult to generalize to other areas^8^. There are therefore increasingly many efforts towards large-scale multi-catchment studies^9–12^. This trend toward large-scale studies, and therefore large-scale databases, should, however, balance breadth and depths^13^. Another reason to move toward large-scale studies is the challenge of a changing climate. Most early water resources climate change impact studies focused on single catchments. We now know that climate variability is region dependent and scale dependent^14,15^. Hydrological climate change impact studies should therefore be conducted at least at the regional scale and on catchments of different sizes to better understand the impact of internal variability and amplification of precipitation extremes^16^ as a function of the spatial scale.

**Fig. 1:**
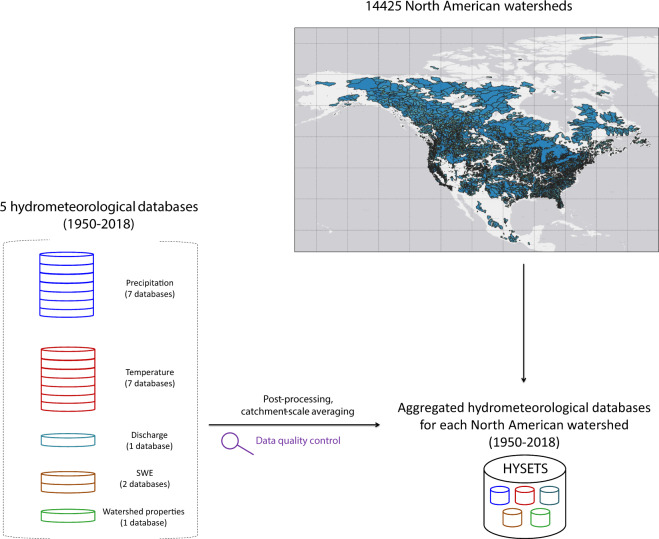
Schematic view of HYSETS database. 14425 North American (NA) watersheds are included in the database. This NA database includes data over the 1950–2018 period: (1) daily precipitation and minimum and maximum temperature products from 7 data sources; (2) hydrometric gauging station discharge time-series from one data source per country (Canada, Contiguous U.S., Mexico); (3) SNODAS and ERA5-Land snow water equivalent; and (5) watershed properties from PAVICS-Hydro (area, elevation slope, land use, soil properties and other physiographic information).

Many efforts have been put into building relevant large-scale hydrometeorological databases. The Global Runoff Data Center^17^ which has been operational since 1988, was a key effort in trying to regroup many national runoff databases. It is, however, a simple repository of streamflow gauges records with limited metadata. In most countries, agencies in charge of streamflow monitoring are typically different entities than the ones in charge of weather and climate monitoring. It is therefore not surprising that most current catchment-based hydrometeorological databases originate from university and research center efforts. The CAMELS framework for the US^12^ which was extended to Great-Britain^18^ and Chili^19^, as well as the CANOPEX database in Canada^20^ are examples of such efforts. These databases, however, contain a relatively modest number of watersheds (<800) and only use a single weather forcing dataset. A high-resolution global DEM was combined with many global and national streamflow databases to provide the Global Streamflow Indices and Metadata Archive^21,22^. This allowed for the extraction of a significantly larger number of catchments as well as global coverage. GSIM does not, however, provide streamflow or forcing data. The dataset presented in this paper builds on those efforts to provide a complete North American (Canada, United States, Mexico) hydrometeorological database for 14425 catchments, as shown in Fig. 1.

## Methods

The HYSETS database contains a multitude of datasets that, when combined, provide a rich and comprehensive environment for testing in various applications such as in hydrological modelling. Four categories of data are combined to provide this sandbox environment: Hydrometric, watershed delineation, meteorological and physiographic data. The methods to extract, validate and combine these sources of data are presented according to each category.

### Hydrometric data

The daily hydrometric data were collected independently for the three countries covered in this database, namely Canada, the United States and Mexico. The Canadian hydrometric data were provided through the Environment and Climate Change Canada (ECCC) Water Survey Canada (WSC) National Water Data Archive (HYDAT), available at: https://collaboration.cmc.ec.gc.ca/cmc/hydrometrics/www/. The data are downloadable in a single Microsoft Access table and include station metadata, such as station location, drainage area and flow regime, as well as the actual daily flow data. A filter was applied to select only the stations whose flow regime is natural, i.e. is not regulated by man-made structures over the study period. This was performed to ensure that the hydrometric data and meteorological data were as naturally correlated as possible.

Hydrometric data for the United States were collected from the United States Geological Survey (USGS) National Water Information Service (NWIS) web portal^23^, available at: https://waterdata.usgs.gov/nwis/uv/?referred_module=sw. Data were batch-downloaded and processed to obtain full time-series for each station. While some metadata is made available such as drainage area, station coordinates and statistics on historical flow, there is no information on the flow regime or on the presence of regulation structures. An alternative method to filter regulated structures was thus devised. To determine which sites were regulated or were affected by regulation, the streamflow dataset was cross-checked against the peak flow statistics database from NWIS. Stations whose peaks were influenced by regulation works at least once in the archive were removed from the HYSETS dataset. Therefore, all regulated, or partly regulated stations were excluded to keep the data as close to natural as possible. While this method seems to have produced the desired results, it is possible that the filtering method is not perfectly accurate and as such, users should verify individual hydrographs if any doubts arise on the river’s regulation status.

Finally, hydrometric data in Mexico were collected from the “Banco Nacional de Datos de Aguas Superficiales” (BANDAS) produced and maintained by the National Water Commission (CONAGUA) from Mexican Ministry of Environment. The data were downloaded from the BANDAS web portal and was filtered according to data quality (visual inspection, hydrological model calibration performance) and time series length. The BANDAS data are available at: www.conagua.gob.mx/CONAGUA07/Contenido/Documentos/Portada%20BANDAS.htm.

Metadata is available for every station in the database and includes station coordinates, drainage area and information about the presence/absence of regulation structures. The stations included in the HYSETS database are all located on basins exempt from any regulation structures. Finally, a filter was applied to ensure that all stations had at least one year of recorded streamflow data. All hydrometric data were converted into units of cubic meters per second (m^3^/s).

### Watershed delineation

The watershed delineation boundaries are a critical component of the dataset as all meteorological data need to be extracted according to those limits for each watershed. For most of the watersheds, the water management agencies provide the official boundaries directly in the form of a shapefile or geodatabase. The Canadian data are available at: http://donnees.ec.gc.ca/data/water/products/national-hydrometric-network-basin-polygons/?lang=en and the United States boundaries are available at the following website: water.usgs.gov/GIS/metadata/usgswrd/XML/streamgagebasins.xml.

The drainage area was made available for most hydrometric gauges by the water management agencies that collate and curate those sources of data. However, a filter was applied to remove all stations that did not have an official drainage area value at the hydrometric gauge, as the value is key in determining if the watershed bounds are acceptable or not. The drainage areas were validated using the watershed delineation boundaries as described above in the geospatial analysis software QGIS 3.4. However, in some instances, the water management agencies did not provide watershed boundary files as they had not been produced or made available publicly. In those cases, estimated watershed contours were taken from the Global Streamflow Indices and Metadata (GSIM) project^21,22^ where available. For catchments where GSIM boundaries were kept for the data extraction a flag (“flag_GSIM_boundaries”) was set to 1 to inform users that the boundaries are from GSIM and not from the official agencies. The GSIM-derived area is also identified in those cases in the dataset, under the “Drainage_Area_GSIM_km2” heading. For catchments smaller or equal to 50 km^2^ in size according to the official gauge, a bounding box equal to the surface area around the catchment outlet was provided as the contour of the catchment as at those scales catchment delineations are difficult due to the resolution and hydrometric gauge accuracy. These catchments are represented by the “flag_artificial_boundaries” indicator in the dataset files. Furthermore, weather data and other catchment attributes are coarser than the area of the catchments in most cases.

In the HYSETS database, all boundaries are provided in a WGS84-projected ESRI shapefile and include the following properties: Watershed ID (to link to the data in the netCDF files), source of the data, name of the hydrometric station, official ID of the hydrometric station and the flag to identify if the boundaries were derived from GSIM. All drainage areas are in km^2^.

### Meteorological data

The HYSETS database contains meteorological data from five sources. The following sections detail the methods applied to integrate the data into the database at the catchment scale. All precipitation data are provided in millimetres per day (mm/d), and temperature values are in degrees Celsius (°C).

#### Station data

Three weather station products were used to cover the North-America domain: The Environment and Climate Change Canada (ECCC) weather stations for Canada, available at: https://climate.weather.gc.ca/, the Global Historical Climate Network Daily (GHCND) station database^24,25^ for the United States and Mexico, available at: https://www.ncdc.noaa.gov/ghcnd-data-access and the station-based serially complete dataset for North America (SCDNA)^26^, available at: https://zenodo.org/record/3735534. The ECCC and GHCND datasets were combined to provide a North American weather station dataset for raw observations with a potential incomplete coverage for the desired 1950–2018 period. The SCDNA was also added to provide another dataset of stations with complete records between the 1979–2018 period.

The daily historical ECCC weather data were downloaded from the ECCC web portal for the years 1950–2018. Data include daily precipitation, maximum and minimum temperature for over 8578 stations across Canada, with varying levels of data completeness and record length. While the GHCND database contained Canada’s weather stations as well, the ECCC database was more complete and was preferred. Over Mexico, a total of 5249 precipitation and 5071 temperature stations are available in the GHCND dataset. Similarly, there are 55693 precipitation and 16011 temperature stations across the United States. While these stations undergo some quality control (QC), both data with and without QC were extracted and provided in the HYSETS database.

These stations were combined into a 69520-station dataset for precipitation and 29960-station dataset for temperature. The extraction process was performed twice for station data: Once using the quality controlled GHCND database and another using all available data, even the non-quality-controlled data.

The SCDNA provides daily precipitation, maximum and minimum temperature for 27280 stations over North America. A strict quality-control was performed on the original station data by the SCDNA authors. Missing data were infilled/reconstructed using information from neighbouring stations as well as three reanalyses products (ERA5, JRA-55 and MERRA-2). Strategies based on quantile mapping, statistical interpolation and machine learning were used to implement the corrections. Overall, this dataset was shown to provide a better agreement to station observations compared to four gridded products.

All three station datasets (ECCC/GHCND with and without QC and SCDNA) were weighted separately using Thiessen polygons for each watershed. The stations contributing to the Thiessen polygon calculation were those located within an artificial boundary defined as the real watershed boundaries extended by a buffer of 1° of latitude and longitude. This step was performed in order to exclude stations that would be too distant to represent the watershed conditions. Due to the highly variable nature of gauge-data quality and station longevity in the case of the ECCC/GHCND combined datasets, Thiessen polygons were computed for each day, using the available data for each day. Therefore, there are some discontinuities when a station is added or removed, or when a station temporarily has no record for a given period of time. There are also cases where no data at all were available for a given period. In those cases, the meteorological data fields are set to NaN, and the user is encouraged to replace those data using other means (either manual replacement or replacement with one of the gridded products as described below). The 1950–1978 period for the SCDNA dataset was also set to NaN.

All three sets of catchment-averaged data are available in the HYSETS database, opening the possibility of performing quantitative assessments of the impacts of using more (but perhaps less reliable) meteorological data in impact studies.

#### Natural resources canada gridded climate data for canada

The Natural Resources Canada (NRCan) gridded climate data product was made available from Natural Resources Canada’s Canadian Forest Service and covers the entirety of Canada up to approximately 84°N latitude^27–29^. It includes daily precipitation, maximum and minimum temperature data on a daily scale on a ~10 km spatial grid. Data cover the period 1950–2010 inclusively. Data points falling within the catchment boundaries were averaged to obtain a single time-series of continuous data as the NRCan dataset contains no missing data. When catchments were too small to contain a data point, the closest data point to the catchment centroid was used to populate the time series for that watershed. Information on the NRCan dataset can be found at the following website: https://cfs.nrcan.gc.ca/projects/3/4.

#### Livneh gridded climate data for continental USA, Mexico and southern Canada

The Livneh database includes interpolated precipitation and temperature data on a regular 0.0625 × 0.0625° grid over the continental United States, Mexico and southern Canada^30,31^. The data cover the period 1915–2015, although only the portion 1950–2015 was used in this dataset. It includes daily precipitation, maximum and minimum temperatures for the entire period without any missing data. Some discontinuities are present at the United States/Mexico border as station density and quality differ and influence the interpolation process. The same is also present but at a smaller scale on the United States/Canada border. The catchment-averaging process was the same as for the NRCan dataset. The Livneh data were provided by the NOAA/OAR/ESRL PSD, Boulder, Colorado, USA, from their Web site at https://www.esrl.noaa.gov/psd/.

#### ERA5 and ERA5-Land reanalyses

The ERA5^32^ and ERA5-Land^33^ reanalyses are hourly products developed by the European Center for Medium-Range Weather Forecasting (ECMWF). The reanalyses provide estimates of a multitude of hydrometeorological and atmospheric variables including precipitation and temperature on regular grids covering the entire surface of the Earth. ERA5 was first implemented with a 0.25° x 0.25° spatial resolution and data are available from 1979–2019, although the HYSETS database stops in 2018 to remain consistent with the other data sources. The ERA5-Land reanalysis is a refined version of ERA5 with a spatial resolution of approximately 9 km. It was driven by the ERA5 reanalysis and a mask was applied such that only land masses are modelled in the refined domain. ERA5-Land covers the period 1981-onwards, and as such the years 1981–2018 are available at present in the HYSETS database.

As both the ERA5 and ERA5-Land products are hourly and data are archived without UTC offsets, it was required to shift the data according to the grid point locations. The longitude of the watershed centroid was used to assess to which time zone it belongs. Based on this time zone, the hourly data were shifted from the same number of hours to realign the daily cycle between 00:00 and 24:00. For instance, a station located in the time zone -7 will have the whole time series shifted by 7 hours to match its proper daily cycle. The ERA5 and ERA5-Land data were downloaded from the Copernicus Climate Data Store, available at: https://climate.copernicus.eu/climate-reanalysis. Note that the HYSETS dataset contains modified ERA5 and ERA5-Land reanalysis data from the Copernicus Climate Change Service Information and that neither the European Commission nor ECMWF is responsible for any use that may be made of the Copernicus Information or Data it contains.

Once the data processing was complete to bring it to the daily scale, the extraction followed the same process as for the NRCan and Livneh datasets, however, for the reanalysis products, the data are available for all watersheds given that reanalyses cover the entire globe.

#### Snow Water Equivalent (SWE) data

Two snow-water equivalent databases are provided in HYSETS. The first is a 9-year (2010–2018) time series of watershed-averaged daily high-resolution (roughly 1 km) data provided by the Snow Data Assimilation System (SNODAS) analysis^34^ available at: https://nsidc.org/data/g02158. The SNODAS data were averaged at the catchment scale using points within the watershed boundaries, or the closest point for the smallest of watersheds that did not contain a point within their limits. SWE data units are millimetres (mm) and represent the value expected on the ground for that day. Any missing values are replaced by NaNs. SNODAS data incorporate multiple sources of data and provide the best possible estimate of SWE at each point location. HYSETS simply averages those values at the catchment scale for users to easily interpret and analyse the data with respect to the rest of the data. SNODAS’ main limitation is that its spatial coverage includes the continental USA as well as the lower portions of Canada below 54°N latitude. This means that many snowy catchments in northern Canada and Alaska are not covered by SNODAS.

The second dataset is the ERA5-Land reanalysis product, which covers the period 1981–2018. It was extracted using the same method as for the ERA5-Land precipitation and temperature data and was also averaged at the daily scale using an hourly UTC offset depending on the time zone. One main advantage of the ERA5-Land reanalysis SWE is that it covers the entire globe and thus is available for all catchments, even those above 54°N that are not covered by SNODAS.

### Physiographic data

One of the strengths of the dataset is the inclusion of a multitude of properties to describe and characterize each of the watersheds. The process is similar for all of the properties, but some variables required slightly more complex operations than others.

The first set of data was based on geographic and topographic properties and was derived from the EarthEnv-DEM90 digital elevation model^35^ available at: https://www.earthenv.org/DEM.html. The process was performed in a meta-software called PAVICS-Hydro (Power Analytics and Visualization for Climate Science - hydrological modelling toolbox) being developed for this purpose, available at: https://pavics-sdi.readthedocs.io/. This set includes mean watershed elevation (meters), slope (degrees), aspect (degrees), Gravelius index (unitless) and perimeter (kilometers). The elevation and perimeter are self-explanatory. The slope is the average slope when considering the individual elevation differences between tiles and can be seen as an indicator of the catchment relief, with higher slopes indicating more mountainous regions. The aspect is the main orientation of the catchment, i.e. where the average slope points towards. The Gravelius index is the ratio of the perimeter of the watershed compared to the perimeter of a circle of the same area. Higher values indicate more elongated or less compact catchments. All the DEM points falling within a watershed boundary were used to compute these characteristics.

The database also provides elevation band data for each catchment in 100-meter intervals. This information was extracted from the EarthEnv 90 m DEM by applying a zonal histogram to a reclassified DEM based on 100-meter intervals in the QGIS software. The data are provided in a separate.csv file “HYSETS_elevation_bands.csv” and represent the percentage of catchment area lying below that elevation. Therefore, the curves are cumulative sums of these areas. This will allow users to provide information to more complex routines and models such as the well-known CemaNeige snow accounting and melt model. The elevation bands can also be used to adjust precipitation rates and temperatures per precipitation bands based on the average catchment elevation and the elevations in the bands, depending on the desired lapse rates and correction methods.

The land use percentages reflect which fraction of the watershed is covered in the different classification categories. The North American Land Change Monitoring System (NALCSM) imagery data from 2010 was used for this purpose^36,37^. NALCMS was developed by Canada, the United States and Mexico to track the evolution of land use over time. A static dataset for 2010 is available and contains 19 land use classes values that were combined to form 7 meta-categories: forests, shrubs, croplands, wetlands, water, urban and permanent snow/ice. For example, coniferous, deciduous and mixed forests were combined into the “forest” category. The original data are available at the Commission for Environmental Cooperation website:

http://www.cec.org/tools-and-resources/north-american-environmental-atlas/north-american-land-change-monitoring-system

For each watershed, the NALCMS raster dataset was queried through the PAVICS zonal statistics toolbox for each of the 19 original categories. The values were then aggregated to the 7 categories used in the HYSETS dataset and the relative fraction of each was computed.

The dataset used to characterize catchment geology is the GLobal HYdrogeology MaPS (GLHYMPS) of subsurface permeability and porosity^38^. This dataset provides quantitative estimates of permeability and porosity below the soil horizon. Catchment averages of these two variables were calculated, by considering the contribution of each spatial polygon being weighted by the fraction of a catchment it covers. The arithmetic mean was used for porosity, but for permeability, the geometric mean was taken. The same process as for NALCMS was performed. However, a pre-processing of the GLHYMPS vector data was performed to transform it to a raster format. The same zonal statistics tools from PAVICS were used to extract the average values of both variables for each catchment. The permeability units are in m^2^ whereas the porosity is archived as a fraction. The GLHYMPS dataset is available at: https://dataverse.scholarsportal.info/dataset.xhtml?persistentId=doi:10.5683/SP2/DLGXYO.

For all the above-mentioned properties, if for any reason the extraction process could not be performed (watershed out of product boundaries, unavailability at a given location) the values are replaced by NaNs and a flag was set in the metadata and properties file.

### Monthly hydrometeorological data

One aspect that must be noted is that the data are all averaged temporally at the daily scale and spatially at the catchment scale. This means that for large catchments, it is possible that the daily data are not very representative of the localized precipitation and runoff events. For this reason, the HYSETS database also includes monthly-aggregated data for all catchments, which will allow evaluating products in terms of bias and mass balance over longer periods. The daily data are still made available for all catchments and the users are invited to consider which timescale is more appropriate for their use-case.

The monthly hydrometeorological data include data from the seven temperature and precipitation data sources as well as for the streamflow. SWE values were not provided at the monthly scale as they are typically not as variable as other variables. All monthly data are combined into a single netCDF file named “HYSETS_2020_monthly_meteorolgical_data.nc”.

## Data Records

All data are available via the Open Science Framework data repository^39^ at: 10.17605/OSF.IO/RPC3W. Metadata detailing watershed properties are presented in a semicolon delimited text file “HYSETS_watershed_properties.txt” and include watershed name, source, centroid coordinates, area, physiographic properties and extraction flags to indicate where values are available. A.csv file containing all elevation band data named “HYSETS_elevation_bands_100m.csv” is also available.

A zip file named “HYSETS_watershed_boundaries.zip” contains the ESRI shapefile containing all watershed boundaries and projection information. Finally, an individual netCDF file was generated for each of the meteorological data sources as described in Table 1.

**Table 1:** Data records for hydrometeorological data.

Filename	Data type and source
**Hydrometeorological netCDF files containing precipitation (pr), maximum temperature (tasmax), minimum temperature (tasmin) and discharge (discharge) data**	
HYSETS_2020_QCstations.nc	Quality controlled station data
HYSETS_2020_nonQCstations.nc	non-Quality controlled station data
HYSETS_2020_SCDNA.nc	Serially complete temperature and precipitation dataset for North America
HYSETS_2020_NRCAN.nc	NRCan gridded interpolated gridded dataset
HYSETS_2020_Livneh.nc	Livneh gridded interpolated gridded dataset
HYSETS_2020_ERA5.nc	ERA5 reanalysis data
HYSETS_2020_ERA5Land.nc	ERA5-Land reanalysis data
**Hydrometeorological netCDF files containing precipitation (pr), maximum temperature (tasmax), minimum temperature (tasmin) and discharge (discharge) at the monthly scale**	
HYSETS_2020_monthly_meteorological_data.nc	Monthly version of the daily data sources.
**Hydrometeorological netCDF file containing only snow water equivalent (swe) and discharge (discharge) data**.	
HYSETS_2020_SNODAS_SWE.nc	SNODAS snow water equivalent dataset
HYSETS_2020_ERA5Land_SWE.nc	ERA5-Land reanalysis SWE data

## Technical Validation

The data were validated in two steps. First, as described in the methods section, filters were applied during the processing to exclude catchments with regulation structures, with poor (or nonexistent) watershed boundaries, too few hydrometric data points and by carefully indicating that some watershed boundaries were extracted from GSIM instead of from an official agency dataset.

**Fig. 2:**
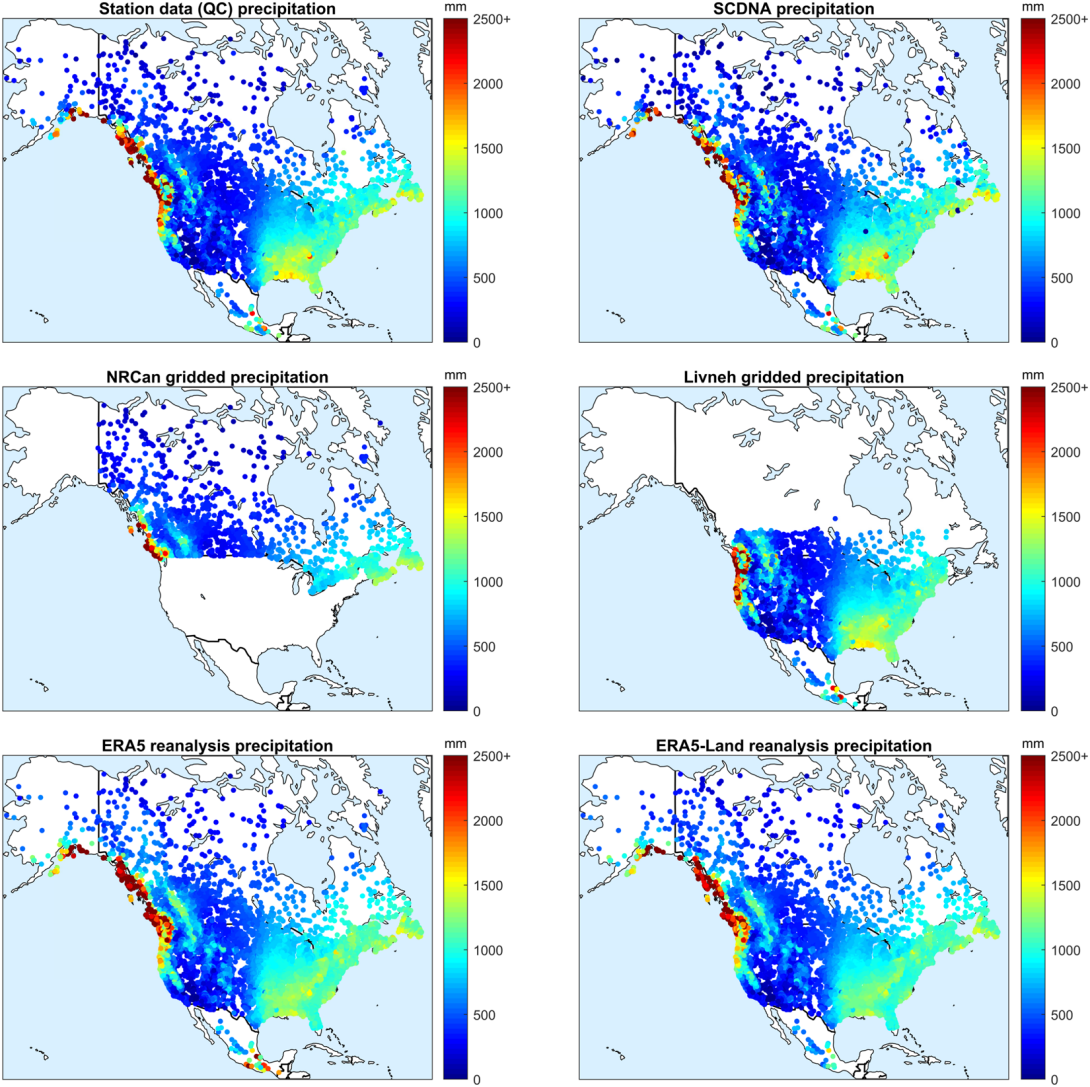
Comparison of average annual precipitation depths in the HYSETS catchments across North America. Precipitation data from the six data sources are displayed in millimetres. The data sources are the station data (top left), SCDNA (top right), NRCan (center left), Livneh (center right), ERA5 (bottom left) and ERA5-Land (bottom right).

Second, the data were displayed on maps to show the similarities and differences between the various datasets and to allow comparison to atlases and other knowledge bases. Figure 2 shows the comparison of the 6 precipitation databases (all except the non-quality-controlled stations). It can be seen that all datasets show the same patterns across North America. Similar comparisons were performed on North America in another study on approximately 3000 catchments and obtained maps that are very similar to the ones in Fig. 2^11^. All datasets clearly show the high west-coast precipitation patterns and the drier Midwest and Canadian prairies.

**Fig. 3:**
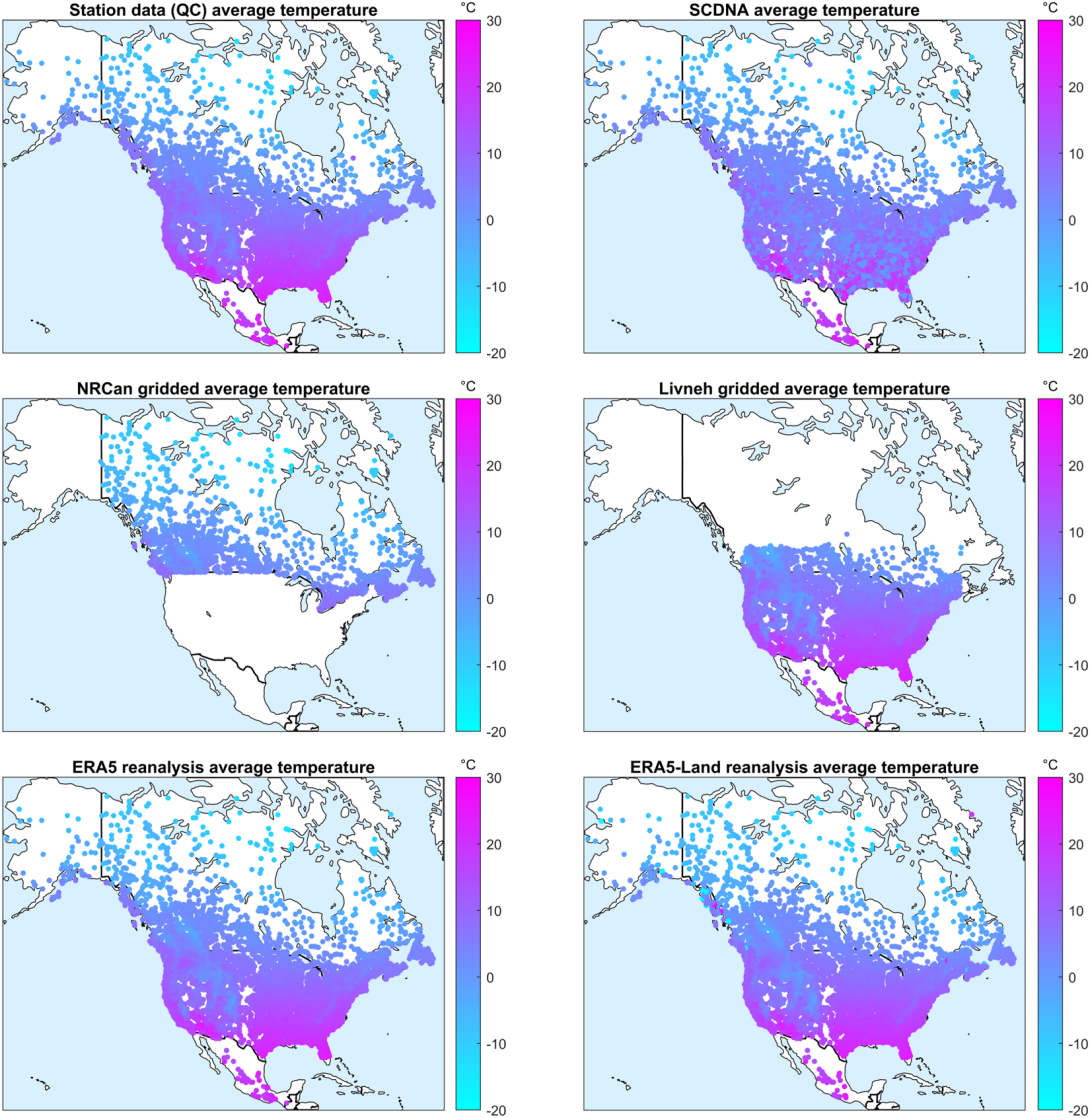
Comparison of average daily temperature in the HYSETS catchments across North America. Temperatures from the six data sources are presented in degrees Celsius. The data sources are the station data (top left), SCDNA (top right), NRCan (center left), Livneh (center right), ERA5 (bottom left) and ERA5-Land (bottom right).

Figure 3 shows a similar comparison for daily average temperature. The north-south gradient as well as the high-elevation gradients in the mountain ranges of the Western United States and Canada are clearly seen. There are some small differences between the different products when observing in detail, which corresponds to the various product data sources. These findings are true for all the temperature datasets in this database.

**Fig. 4:**
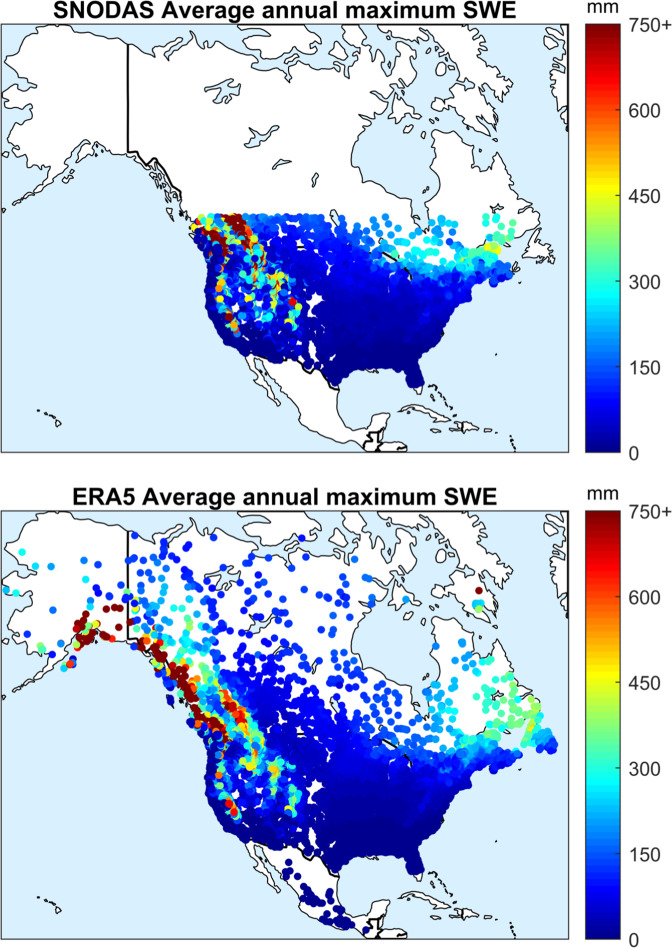
Average annual maximum snow water equivalent amount in the HYSETS catchments across North America. The snow water equivalent is taken from the SNODAS database (top panel) which extends up to 54°N latitude and from ERA5-Land (bottom panel) which covers the entire domain.

Figure 4 presents the average maximum annual SWE amounts over the dataset region. Again, these values represent well the expected values for North America, with no snow in the southern parts of North America and more snow in the North and mountain ranges. Extents are limited by the SNODAS original extents in the top panel and cover the entire domain with the ERA5-Land product in the bottom panel.

**Fig. 5:**
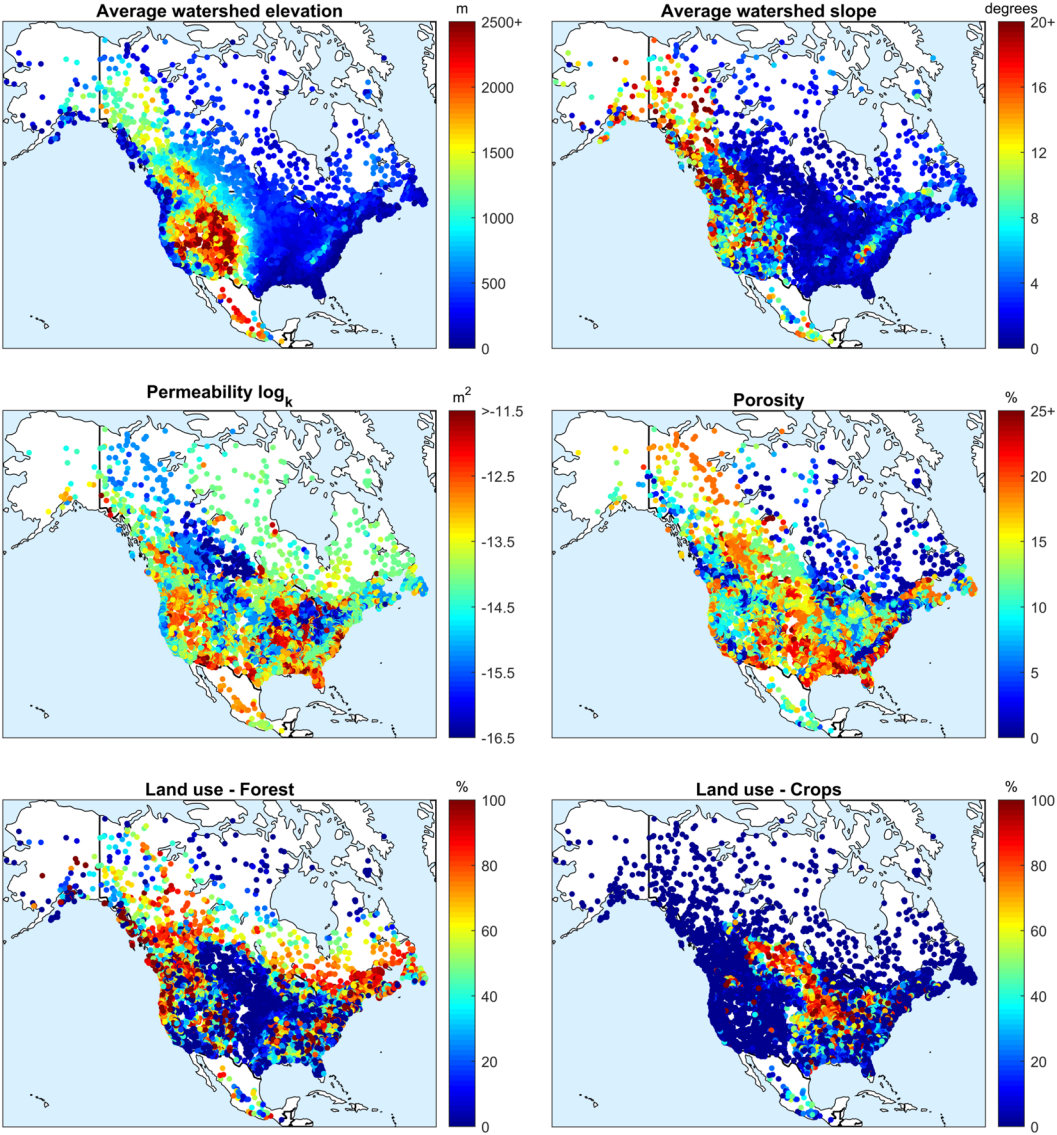
A selection of average watershed properties in the HYSETS catchments across North America. The data presented include elevation (top left), slope (top right), permeability (center left), porosity (center right), forest fraction (bottom left) and cropland fraction (bottom right).

Finally, the watershed properties were analyzed and mapped to obtain a visual confirmation of the various fields. Figure 5 presents watershed-averaged values of elevation, slope, soil permeability and porosity and land use fractions for forests and croplands. It can be seen that elevations map well with the actual topography of North America and that the mountainous regions show higher average slopes, as expected. Porosity and permeability are more variable in nature but reflect the data present in the GLHYMPS subsoil dataset. Finally, land cover fractions are also as expected, with large portions of the United States Midwest having cropland as the dominant land use category and major forested areas in Canada, north-east United States and Western United States. Figure 5 also shows that the Mexican catchments are highly heterogeneous in terms of physiographic attributes^40^.

Finally, the Global Streamflow and Indices Metadata (GSIM) database^21^ found approximately 15750 stations in North America that were of high enough quality to be included. The HYSETS database independently found 14425 catchments of high quality from a total number of over 25000 initially available. This gives confidence that the verification methods and filters put in place allowed a similar cut-off to those in the GSIM database.

## Usage Notes

The HYSETS database contains multiple sources of data that can be used to evaluate their relative performance and lead to multi-component analyses. For example, precipitation data from ERA5, ERA5-Land and SCDNA can be compared from a meteorological point of view, considering that SCDNA integrates multiple sources of precipitation data including stations and reanalysis data for infilling. Validation through comparison with station-based (precipitation and temperature) data can also be performed over all of North America, including in highly heterogeneous climates. Performance assessment through hydrological model calibration can be undertaken, and many combination assessments can be performed through multi-input experiments^41^. The performances of the Livneh and NRCAN gridded datasets can also be compared in southern Canada, while the impact of the spatial resolution refinement from ERA5 to ERA-Land on precipitation outputs and on hydrological modelling results can be assessed. Users could want to use the SNODAS and ERA5-Land SWE values to constrain hydrological models during the calibration process and to validate each other in hydrological modelling applications.

As the database covers a wide spatial domain with a great diversity in watershed characteristics, the elevation, slope, land use, porosity and permeability data can be used in large-scale regionalization studies, to better assess the performance of various methods for streamflow prediction in ungauged basins. The numerous nested basins can also help in developing transfer functions from gauged to ungauged sites. The wide range of watershed sizes allows exploring and testing different hypotheses on hydrological processes modelling across spatial scales.

The database also has possible usages for climate change impact studies. The multiple temperature and precipitation datasets represent a diversity of reference data for bias correcting global or regional climate model outputs. Since the database covers a time period of up to almost 70 years, it allows performing trend analyses and investigating recent past changes in hydroclimatic variables in different regions of North America. The considerable diversity of physiographic characteristics and hydroclimatic conditions also creates a vast playground for studying, for instance, hydrological model parameters sensitivity and variability as well as hydrological processes and the impact of watershed characteristics on modelling those processes.

The dataset currently covers the period 1950–2018, and it is planned that it will be updated regularly as new datasets are created and made available. We expect to update the database approximately once per year, or more often when relevant new datasets are made publicly available.

## Data Availability

The codes used to generate this dataset can be shared by contacting the authors. Three main types of codes were used: 1. Codes to download and extract the data. Most of the data were downloaded via requests to the public-facing servers using their API (WSC and USGS hydrometric data, for example) and through their data dissemination mechanisms (ECMWF ERA5 and ERA5-Land). Other data were downloaded from FTPs and thus did not require specific codes (SNODAS snow water equivalent data, GHCND station data). The “ghcnd_access” toolbox^42^ was used to extract the GHCND station daily data from the.dly file format into a more accessible format. The “usgsrdbread.m” MATLAB script written by Kelly Kearney was used to extract the USGS streamflow data to the MATLAB format. The code is available here: https://github.com/kakearney/usgsrdbread-pkg. 2. Codes to process data at the catchment scale. These codes were mainly a) to perform Thiessen polygons to extract meteorological data from stations at the catchment scale, b) average netCDF file outputs at the catchment scale (or take the closest point), and c) extract the physiographic data from the land-cover, elevation and soil properties raster images. PAVICS was used in the creation of this database, however any GIS software could be used to extract the zonal statistics from the raster data for each catchment. 3. Codes to ensure data quality and producing the final output formats. These codes are a set of checks to ensure that missing data was handled properly (set to NaN rather than 0), that catchments outside of a particular dataset’s boundaries were excluded for that product, and that basins with smaller areas than 1 km^2^ or were misrepresented by the official agencies were excluded. For example, the USGS NWIS contains data for the island of Guam, which was excluded using these validation tools. The data production tools are simple aggregation tools that convert the data from the MATLAB processing software to more convenient netCDF files.
